# Peripheral and local predictive immune signatures identified in a phase II trial of ipilimumab with carboplatin/paclitaxel in unresectable stage III or stage IV melanoma

**DOI:** 10.1186/s40425-017-0290-x

**Published:** 2017-11-21

**Authors:** Rahima Jamal, Réjean Lapointe, Eftihia Cocolakis, Paméla Thébault, Shirin Kazemi, Jennifer E. Friedmann, Jeanne Dionne, Jean-François Cailhier, Karl Bélanger, Jean-Pierre Ayoub, Huy Le, Caroline Lambert, Jida El-Hajjar, Léon C. van Kempen, Alan Spatz, Wilson H. Miller

**Affiliations:** 1 Hôpital Notre-Dame, Centre de recherche du CHUM, Centre hospitalier de l’Université de Montréal, Montréal, QC Canada; 2 Centre de recherche du CHUM, Institut du Cancer de Montréal, Université de Montréal, Montréal, QC Canada; 3 Segal Cancer Center, Jewish General Hospital, Rossy Cancer Network, McGill University, 3755 Côte-St-Catherine, suite E670, Montreal, Québec Canada; 4 Department of Pathology, Molecular Pathology Center, Jewish General Hospital, McGill University, Montreal, QC Canada

**Keywords:** Melanoma, Ipilimumab, Chemotherapy, Chemokines, Immune score, B lymphocytes, PD-1, Predictive Biomarkers, Immunotherapy

## Abstract

**Background:**

Checkpoint blockade with ipilimumab provides long-term survival to a significant proportion of patients with metastatic melanoma. New approaches to increase survival and to predict which patients will benefit from treatment are needed. This phase II trial combined ipilimumab with carboplatin/paclitaxel (CP) to assess its safety, efficacy, and to search for peripheral and tumor-based predictive biomarkers.

**Methods:**

Thirty patients with untreated unresectable/metastatic melanoma were treated with ipilimumab and CP. Adverse events (AEs) were monitored and response to treatment was evaluated. Tumor tissue and peripheral blood were collected at specified time points to characterize tumor immune markers by immunohistochemistry and systemic immune activity by multiplex assays and flow cytometry.

**Results:**

Eighty three percent of patients received all 5 cycles of CP and 93% completed ipilimumab induction. Serious AEs occurred in 13% of patients, and no treatment-related deaths were observed. Best Overall Response Rate (BORR) and Disease Control Rate (DCR) were 27 and 57%, respectively. Median overall survival was 16.2 months. Response to treatment was positively correlated with a higher tumor CD3^+^ infiltrate (immune score) at baseline. *NRAS* and *BRAF* mutations were less frequent in patients who experienced clinical benefit. Assessment of peripheral blood revealed that non-responders had elevated baseline levels of CXCL8 and CCL4, and a higher proportion of circulating late differentiated B cells. Pre-existing high levels of chemokines (CCL3, CCL4 and CXCL8) and advanced B cell differentiation were strongly associated with worse patient overall survival. Elevated proportions of circulating CD8^+^/PD-1^+^ T cells during treatment were associated with worse survival.

**Conclusions:**

The combination of ipilimumab and CP was well tolerated and revealed novel characteristics associated with patients likely to benefit from treatment. A pre-existing systemic inflammatory state characterized by elevation of selected chemokines and advanced B cell differentiation, was strongly associated with poor patient outcomes, revealing potential predictive circulating biomarkers.

**Trial registration:**

Clinicaltrials.gov, NCT01676649, registered on August 29, 2012.

**Electronic supplementary material:**

The online version of this article (10.1186/s40425-017-0290-x) contains supplementary material, which is available to authorized users.

## Background

Metastatic melanoma has been historically one of the most treatment-resistant human malignancies, with a median survival of 6–9 months with standard chemotherapy [1]. Recently, major strides in the treatment of advanced melanoma were achieved with the approval of two classes of therapies, targeting the MAPK pathway and immune checkpoints. Ipilimumab, a fully humanized monoclonal antibody targeting cytotoxic T-lymphocyte-associated protein 4 (CTLA-4) and a negative regulator of T-cell activation, was the first immunotherapeutic agent shown to prolong survival in this disease [2, 3]. Pivotal studies with ipilimumab demonstrated increased overall survival (OS) as a single agent (10.1 months) and in combination with DTIC (11.2 months). Combining ipilimumab with other treatment modalities to increase its efficacy and searching for predictive biomarkers have been areas of intense research [4–9]. After failure of immune checkpoint inhibitors or in patients progressing rapidly after BRAF and MEK inhibitors, chemotherapies, such as carboplatin and paclitaxel (CP), are still used to treat rapidly growing metastatic melanoma.

Previous studies have demonstrated a role for chemotherapeutic agents in promoting anti-tumor immunity through several mechanisms. Platinum compounds have been found to induce immunogenic cell death [10, 11], a functionally distinct type of apoptosis that elicits tumor-specific cognate immune responses [12]. Preclinical studies show tumor cells treated with taxanes or platinum compounds lead to increased cytotoxic T-cell activation [13–15]. Paclitaxel has also been implicated in direct inhibition of myeloid-derived suppressor cells (MDSCs), a heterogeneous population of cells that are defined by their myeloid origin, immature state and ability to potently suppress T cell responses [16]. Addition of chemotherapy has been shown to enhance anti-CTLA-4 antitumor activity in animal models [17]. Thus, there is a good rationale to combine immune checkpoint inhibitors, such as ipilimumab, with platinum compounds like carboplatin and taxanes such as paclitaxel.

Safety and anti-tumor efficacy of this combination was evaluated. This study also sought to prospectively identify cellular and/or molecular predictive biomarkers.

## Methods

### Study design and treatment

Eligible patients were men and women ≥ 18 years with a histologic diagnosis of untreated, unresectable Stage III/Stage IV melanoma. Prior treatment with BRAF inhibitors in the metastatic setting was permitted. Patients had measurable or evaluable disease, Eastern Cooperative Oncology Group (ECOG) performance status ≤ 1 and adequate bone marrow, liver, and renal function at study entry. Patients with symptomatic brain metastases, autoimmune disease, peripheral neuropathy ≥ Grade 2, prior treatment with a CD137 agonist or a CTLA-4 agonist/inhibitor, chronic use of immunosuppressive drugs or of systemic corticosteroids were excluded.

Treatment consisted of carboplatin (AUC 6)/paclitaxel (175 mg/m^2^) × 5 cycles and ipilimumab (3 mg/kg × 4 cycles) every 3 weeks. Each CP treatment was preceded by dexamethasone premedication. Two different dosing schedules were used, patients were randomly assigned in a 1:2 scheme to receive ipilimumab either concurrently (Arm A, 10 patients) with CP or sequentially 1 week apart (Arm B, 20 patients), starting at Cycle 2. Patients were stratified according to previous treatment with a BRAF inhibitor. Physicians had the option of retreating patients with either ipilimumab alone or in combination with CP if they experienced disease progression following objective response (PR, CR) or SD lasting for 3 months or more. Supportive measures and/or secondary prophylaxis were allowed for CP induced hematologic toxicity.

The primary objective was to determine the safety and tolerability of two schedules of ipilimumab in combination with CP. Secondary objectives were to determine putative early cellular and/or molecular biomarkers for therapy response; to measure anti-tumor efficacy (OS, overall response rate (ORR), PFS, and clinical benefit rate (CBR; ORR + stable disease (SD) ≥ 24 weeks)) by irRC and mWHO response criteria [18].

Response evaluations (progressive disease (PD), stable disease (SD), partial response (PR), complete response (CR)) were defined according to mWHO and irRC guidelines [18]. mWHO results are available in Additional file 1: Figure S1 and Additional file 2: Table S3. Tumor assessments were performed at screening, weeks 8, 16, 24 and approximately every 12 weeks thereafter. Safety assessments included vital signs, physical examinations, laboratory tests, and adverse event reporting (AEs). AEs were graded using the Common Toxicity Criteria for Adverse Events, version 4.0. AEs were recorded from the first study medication dose. SAEs were recorded following consent signature until 70 days from discontinuation of investigational products.

The study was conducted in accordance with the Declaration of Helsinki of 1975. The protocol was approved by local ethics committees. All participants gave written informed consent for the study.

### Correlative studies

DNA was extracted from formalin-fixed and paraffin-embedded tissue sections containing at least 30% tumor cells (cobas®, Roche) collected at screening. Mutation analysis of *BRAF* V600 (cobas® kits, Roche) was performed on a cobas® z480 instrument and confirmed by sequencing (Sanger). For *NRAS*, mutation analysis was performed using the *NRAS* Mutation detection kit (EntroGen) on an LC480 platform (Roche). All manipulations were performed according to College of American Pathologists-compliant standard operating procedures of the Jewish General Hospital Molecular Pathology Centre.

Immunohistochemistry with Ventana Benchmark antibodies towards CD3 (clone 2GV6), CD4 (clone SP35), CD8 (clone 1A5) and PD-L1 (clone SP142) was performed according to standard automated protocols and quantified in agreement with REMARK guidelines [19]. PD-L1 expression was assessed semi-quantitatively as previously described [20]. The immune score was obtained from semi-quantitative prevalence of CD3^+^ cells noted as absent (0), focal (1), moderate (2) or severe (3) as previously described [21].

Blood samples were collected from patients at baseline, weeks 1, 2, 4, 7, 10, 13, 16, 24 and at the end of study. Blood was also obtained from six normal donors (ND). Peripheral blood collection and processing were done as previously described [22]. PBMCs suspended in human serum albumin solution were stored at −80 °C. Cell surface staining was performed as described previously [22, 23] using antibodies (BD Biosciences) listed in Additional file 2: Table S6. Dead cells were excluded with the *Live/Dead Fixable Dead Cell* kit (Invitrogen). Flow cytometry data were acquired using a BDFortessa instrument (BD Biosciences) and analyzed using FlowJo software (Tree Star). Peripheral soluble cytokines/chemokines/soluble receptors (Additional file 2: Table S7) were measured from cryopreserved plasma. All kit components from V-plex Ultra-Sensitive kit (Meso Scale Discovery) were processed as per the manufacturer’s instructions. Electroluminescent data were analyzed with a four-parameter logistic curve fit using MSD Discovery Workbench.

IgG, IgA and IgM concentrations were determined in a standard ELISA as previously previously described [14]. Baseline values from patients and from the normal donors were inside the accepted ranges of reference values for clinical diagnostic purposes (total IgG, 7.0–16.0 g/l; total IgA, 0.7–4.0 g/l; total IgM, 0.4–2.3 g/l).

### Statistical analyses

This study was designed as a pilot study on a limited number of patients to gain insight on safety and efficacy of the combination of ipilimumab and CP and identify putative biomarkers associated with response. Patients and disease characteristics were analyzed using descriptive statistics and expressed as either relative frequency (percentages) for discrete variables or median for continuous variables. Comparisons between patient response groups at baseline and throughout treatment were evaluated by ANOVA. Correlations between mutation and tumor immune profiles and best overall response (BOR) were determined using Chi-square and Pearson correlation tests. Effect of groups and time points and their interaction in blood samples were assessed with linear mixed effect models, multiple comparisons specified in figure legends were done where interactions were not significant. Univariate cox proportional hazards regression analyses were used to assess correlation of variables with OS. Chemokine, Bm2 and eBm5 + Bm5 levels were logarithmically transformed prior to regression analyses. OS Kaplan–Meier curves were compared by the log-rank test. Cutoff thresholds defining high or low levels were established from the mean value of a given variable from all patients. A *P* value < 0.05 was considered statistically significant. Statistical analyses were performed with SPSS 25 software (SPSS Inc.), GraphPad Prism 6.01, and R software i386 3.0.2 [24].

## Results

### Patient selection

Thirty patients were enrolled onto the study from December 2012 until October 2013 (Table 1, Additional file 2: Table S1). Fifty-seven percent of patients had M1c disease, and 33% of patients had LDH levels higher than the upper limit of normal at baseline. Seventeen percent of patients had received prior adjuvant therapy. Nine patients had a *BRAF* mutated melanoma, of whom six had been treated with a BRAF inhibitor in the metastatic setting. Eighty-three percent of patients received all five cycles of CP, and 93% received all four doses of ipilimumab. One patient did not receive ipilimumab because of early clinical progression. Six patients meeting the retreatment criteria were retreated with either ipilimumab/CP (2/6) or ipilimumab alone (4/6).

**Table 1:** Demographic and baseline characteristics of the patients

Patient Characteristics	ALL patients (Number (percent))
Total no. of patients	30 (100)
Median age, years (range)	55 (26–74)
Sex	
Female	8 (27)
Male	22 (73)
Metastatic stage (n)	
M0	1 (3)
M1a	6 (20)
M1b	6 (20)
M1c	17 (57)
Lactate dehydrogenase	
≤ ULN^a^	20 (67)
> ULN	10 (33)
ECOG	
0	19 (63)
1	11 (37)
Primary site	
Cutaneous	24 (80)
Mucosal	2 (7)
Ocular	3 (10)
Unknown primary	1 (3)
*BRAF* status	
*BRAF* mutated (V600E)	9 (30)
*BRAF* wild type	21 (70)
Prior therapies**	
Prior adjuvant therapy	5 (17)
Prior therapy with a BRAF inhibitor	6 (20)
Brain metastases	
Patients without brain metastases	26 (87)
Patients with brain metastases	4 (13)

^a^ULN denotes upper limit of the normal, ** One patient (3%) received 2 weeks a of Temozolomide

### Toxicity

The most common Grade 3–4 adverse events found in ≥ 10% of patients were diarrhea, neutropenia and thrombocytopenia (Table 2, Additional file 2: Table S2). The rate of febrile neutropenia was 7% (2/30). Four patients (13%) had treatment-related SAEs. Two patients discontinued chemotherapy because of drug-related AEs (Grade 3 ALT and Grade 4 neutropenia). Four patients had infusion related reactions to paclitaxel. Grade 3–4 AEs related to ipilimumab were found in 13% of patients. Twenty percent (6/30) of patients received steroids for immune-related adverse events (irAEs): one patient with Grade 3 autoimmune colitis, two patients with Grade 3 diarrhea, two patients with Grade 2 endocrinopathy, and one patient with Grade 2 rash. One patient discontinued ipilimumab because of an irAE (Grade 3 autoimmune colitis) during the re-treatment period. No drug-related deaths were reported. Adverse events were comparable between treatment arms (Additional file 2: Table S2).

**Table 2:** Adverse events

	ALL Patients (*n* = 30)	
	Total	Grade3/4/5
All adverse events		
Any event	30 (100)	21 (70)
Hepatotoxicity		
high ALT	4 (13)	2 (7)
Gastrointestinal Disorders		
nausea	21 (70)	1 (3)
vomiting	14 (47)	0
diarrhea	23 (77)	3 (10)
*C. difficile* colitis	1 (3)	1 (3)
Electrolyte		
hypophosphatemia	4 (13)	2 (7)
hypokalemia	6 (20)	1 (3)
hypomagnesemia	8 (27)	1 (3)
Hematological		
anemia	4 (13)	2 (7)
febrile neutropenia	2 (7)	2 (7)
neutropenia	8 (27)	5 (17)
thrombocytopenia	6 (20)	3 (10)
Nervous System		
seizure	2 (7)	1 (3)
vasovagal reaction	1 (3)	1 (3)
Infection		
pneumonia	1 (3)	1 (3)
Constitutional		
fatigue	17 (57)	2 (7)
Vascular Disorder		
pulmonary embolism	1 (3)	1 (3)
Immune-related adverse events		
Any event	26 (87)	4 (13)
Gastrointestinal Disorders		
diarrhea	11 (37)	2 (7)
vomiting	6 (20)	0
autoimmune colitis	1 (3)	1 (3)
Endocrine		
hypothyroidism	1 (3)	0
pituitary disorder	1 (3)	0
Skin Disorders		
rash	7 (23)	0
pruritus	9 (30)	0
urticaria	1 (3)	0
vitiligo	1 (3)	0
erythroderma	1 (3)	0
Constitutional		
fatigue	11 (37)	1 (3)

### Efficacy

**Fig. 1:**
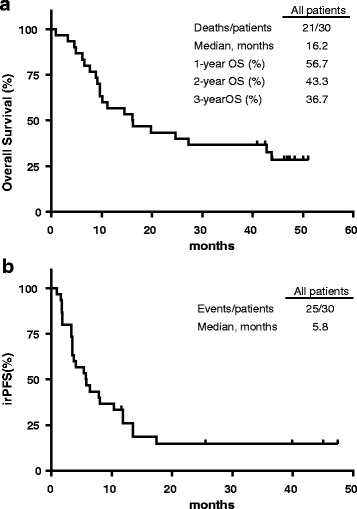
General Kaplan-Meier survival curves – Overall patient survival (**a**) as of February 17th 2017, PFS by immune-related (irRC) (**b**) criteria. Censored data are shown as open boxes on graphs

Patients were followed for up to 46.9 months, with an overall median follow-up of 37.6 months. The median OS was 16.2 months for the entire cohort (Figure 1a). The rate of OS for all patients was 56.7%, 43.3% and 36.7% at 1, 2 and 3 years, respectively. The median PFS was 5.8 months by irRC for all patients (Figure 1b). The best overall response rate (BORR; CR + PR), rate of disease control (DCR; CR + PR + SD), and clinical benefit rate (CBR; CR, PR or SD ≥ 24 weeks) for 30 evaluable patients were 27%, 57% and 43% by irRC, respectively (Table 3). The two mucosal patients had a BOR of PR whereas the three ocular patients all had PD.

**Table 3:** Tumor response (immune related - irRC)

Response	All patients
	*n* = 30
	No. of pts. (%)
irBOR	
irCR	1 (3)
irPR	7 (23)
irSD	9 (30)
irPD	13 (43)
irDCR	17 (57)
irCBR	13 (43)
irBORR	8 (27)

*ir* immune related, *BOR* best overall response, *CR* complete response, *PR* partial response, *SD* stable disease, *PD* progressive disease, *BORR* best overall response rate, *DCR* disease control rate (CR + PR + SD), *CBR* clinical benefit rate (CR + PR + SD ≥ 24 weeks)

### Tumor correlative studies

**Fig. 2:**
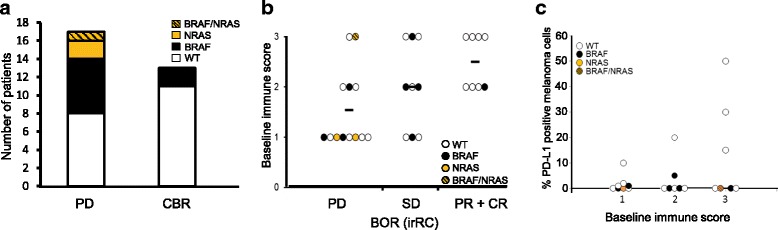
Intra-tumoral biomarkers linked to clinical outcome. **a** Number of patients with a *BRAF* (black), *NRAS* (orange) or *BRAF*/*NRAS* double (orange hatched) mutation in the PD and combined CBR group (CBR; CR, PR or SD ≥ 24 weeks). The frequency of *BRAF* and *NRAS* mutations in PD is significantly higher compared to CBR (Χ^2^ = 4.474, *p* = 0.03) **b** Graphical presentation of the baseline immune score (0: absent; 1: low; 2: moderate; 3: high peri- and intratumor CD3-^+^ cells) per response group (irRC criteria). Mutations type for each sample is indicated: *BRAF* (black), *NRAS* (orange) or *BRAF*/*NRAS* double (orange hatched). A significant correlation between response group and baseline immune score was observed (Pearson correlation 0.487, *p* = 0.01). **c** Baseline immune score (1: low; 2: moderate; 3: high peri- and intra-tumor CD3^+^ cells) and the percentage of PD-L1 expressing melanoma cells. Mutations type for each sample is indicated: *BRAF* (black), *NRAS* (orange) or *BRAF*/*NRAS* double (orange hatched). A trend towards higher PD-L1 expression with increasing baseline immune score was observed (Pearson correlation 0.386, *p* = 0.069). No significant difference between the percentage of PD-L1 positive melanoma cells in tumors with baseline immune score 1 and 3 (Mann Whitney U test *p* = 0.694)

Prevalence of *BRAF* and *NRAS* mutations, immune infiltration and PD-L1 expression in the tumor samples obtained pre-treatment were classified according to response. *BRAF, NRAS* and concurrent *BRAF*/*NRAS* mutations in pre-treatment samples were found in 8/30 (27%, all V600E), 2/30 (7%, both Q61R) and 1/30 (3%, V600E/Q61R) cases, respectively. Mutations in these genes were more frequently observed in PD patients (9/17) compared to the patients that achieved clinical benefit (2/13, *p* = 0.034, Chi-square 4.474, Figure 2a). No correlation between *BRAF* and/or *NRAS* mutation status and OS was observed. Baseline immune score reflecting prevalence of CD3^+^ cells in tumor samples directly correlated with BORR (Pearson correlation 0.487, *p* = 0.01, Figure 2b). Immunohistochemistry analysis of PD-L1 expression at baseline was performed on 22 cases. Twenty-seven percent of cases (6/22) harbored greater than 5% of PD-L1 positive melanoma cells. Lack of PD-L1 expression (<5%) was most frequently observed in irPD (10/12 samples (83%) were PD-L1 negative) compared to SD (1/4 samples (25%) and PR + CR (3/6 samples (50%) were PD-L1 negative) but this did not reach statistical significance (Additional file 2: Table S4). No correlation between the percentage of PD-L1 positive melanoma cells, immune score or BOR or OS was found (Figure 2c, Additional file 2: Table S4, Additional file 2: Table S5).

### Peripheral blood correlative studies: pre-treatment inflammatory status is associated with poor patient outcomes

**Fig. 3:**
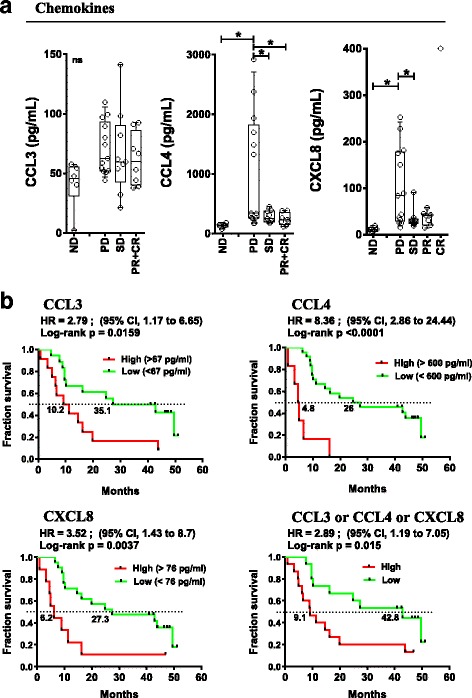
Pre-treatment peripheral chemokine levels associated with response and OS. **a** Baseline (pre-treatment) levels of indicated chemokines according to response status. Each point represents a donor, average is outlined, and error bars represent SEM. ns: not significant; *: *p* < 0.05 from a one-way ANOVA with Bonferroni post hoc test. **b** Kaplan-Meier OS curves for the indicated chemokines stratified by levels above (high) or below (low) the mean level for each chemokine. *P* values were calculated using the log-rank test and are shown on each graph

Pre-treatment soluble and cellular inflammatory markers were studied and correlated with patient outcomes. Baseline levels of 22 peripheral soluble cytokines/chemokines/soluble receptors were measured by multiplex assay (see Additional file 2: Table S7 for the panel used) in the peripheral blood of patients and normal donors (ND). irPD patients had elevated baseline plasma levels of CCL4 and CXCL8 chemokines (1042 and 103 pg/mL, respectively) compared to patients with irSD and irPR who had levels similar to ND (131 and 12 pg/mL, Fig. 3a, *p* < 0.05). No statistically significant differences were detected for other soluble circulating molecules, including CCL3.

Elevated circulating CCL3 (> 67 pg/ml), CCL4 (> 600 pg/ml) and CXCL8 (> 76 pg/ml) correlated with poor OS (Hazard Ratio (HR) =2.8 (95%CI:1.17–6.65), *p* < 0.016; HR = 8.4 (95%CI:2.86–24.44), *p* < 0.0001 and HR = 3.5 (95%CI:1.43–8.7), *p* < 0.004 respectively, Fig. 3b). In patients with an elevation of any of CCL3 or CCL4 or CXCL8, OS was significantly worse (HR = 2.89 (95%CI:1.19–7.05), *p* = 0.015, Figure 3b, bottom right). Pre-treatment levels of CCL3 and CCL4 as continuous variables revealed a statistically predictive significance for OS, HR: 14.14 (95%CI:1.211–165.25), *p* = 0.035 and 5.941 (95%CI:2.06–17.12), *p* = 0.001, respectively (Additional file 2: Table S5). High levels of CCL4 were also associated with levels of LDH above the upper limit of normal (*p* = 0.0179, two-tailed unpaired t test). The data suggest that elevation of the above-mentioned chemokines reveal a pre-existing state of inflammation for patients that predict a poor outcome to therapy with ipilimumab and CP.

Immunophenotyping of PBMCs pre-study treatment revealed no significant changes in the frequency of major immune cell populations (Additional file 1: Figure S2). Analysis of T cell subpopulations suggested that objective responders (irPR + irCR) had a lower proportion of circulating activated CD25^+^CD4^+^ effector T cells (1% of CD25^+^ cells within the effector CD4^+^ T cell gate) compared to patients with irPD, irSD and ND (mean of 3.3% (*p* = ns), 4.5% (*p* = 0.042) and 5.7% (*p* = 0.006), respectively as shown in Additional file 1: Figure S3C. However, increase in this population had no impact on OS (Additional file 1: Figure S3D, Additional file 2: Table S5). Lower levels of additional activation markers on CD4^+^ and CD8^+^ T cell subsets where seen in responding patients (CR + PR) although not statistically significant (Additional file 1: Figure S3A-B).

**Fig. 4:**
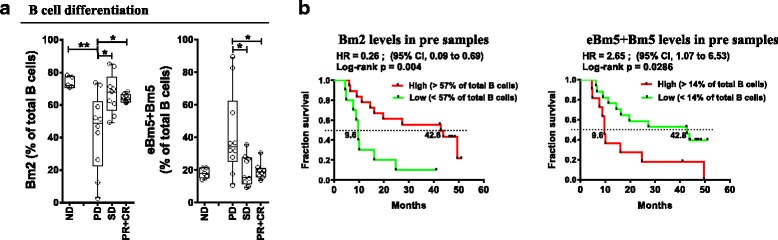
Pre-treatment circulating B lymphocyte differentiation status associated with patient outcomes. **a** Baseline (pre-treatment) percentages of indicated B lymphocytes differentiation status (Bm2 and eBm5 + Bm5), according to the Bm classification (sIgD and CD38; see Additional file 1: Figure S4A for nomenclature and gating strategy), according to response status. Each point represents a donor, average is outlined, and error bars represent SEM. ns: not significant; *: *p* < 0.05; **: *p* < 0.01, from a one-way ANOVA with Bonferroni post hoc test. **b** Kaplan-Meier OS curves for the Bm2 and eBm5 + Bm5 populations, stratified by percentages above (high) or below (low) the mean percentage for each population. *P* values were calculated using the log-rank test and are shown on each graph

B lymphocyte differentiation pattern was assessed from peripheral blood to evaluate pre-existing inflammatory status; more advanced B cell differentiation indicates an ongoing immune response. Indeed, patient response to treatment was associated with their baseline activation pattern of circulating B cells. Circulating B cell subsets from irPD patients displayed a shift towards a late activation/memory phenotype based on surface IgD (sIgD) and CD38 expression [25] (Figure 4a). Specifically, the Bm2 subset (IgD^+^CD38^int^, mature resting B cell) represented 44% of total CD19^+^ cells in irPD patients, compared to 64% in irPR + irCR (*p* < 0.05) and 67% in irSD (*p* < 0.05) patients (ND at 73.9%, *p* < 0.01 when compared to irPD; Figure 4a). The eBm5 + Bm5 subsets (memory B cells, IgD^−^CD38^int/lo^) represented 42.1% in irPD compared to 19% in irPR + irCR (*p* < 0.05) and 19.8% in irSD (*p* < 0.05) patients. ND was similar to responders at 18.2% (Figure 4a). Moreover, pre-existing advanced B cell differentiation had a major impact on OS, where low levels of early differentiated Bm2 (<57%) was strongly associated with poor survival: HR = 0.26 (95%CI:0.09–0.69), *p* = 0.004 (Figure 4b). Conversely, high eBm5 + Bm5 (>14%) levels were strongly associated with better OS: HR = 2.65 (95%CI:1.07–6.53), *p* = 0.029, Figure 4b). An association with OS was also observed when evaluating baseline Bm2 and eBm5 + Bm5 as continuous variables (HR = 0.113 (95%CI 0.023–0.549), *p* = 0.007 and HR = 10.27 (95%CI:1.30–80.96), *p* = 0.027, respectively, Additional file 2: Table S5). These results show that pre-activated B cell differentiation status was associated with poor outcome.

**Fig. 5:**
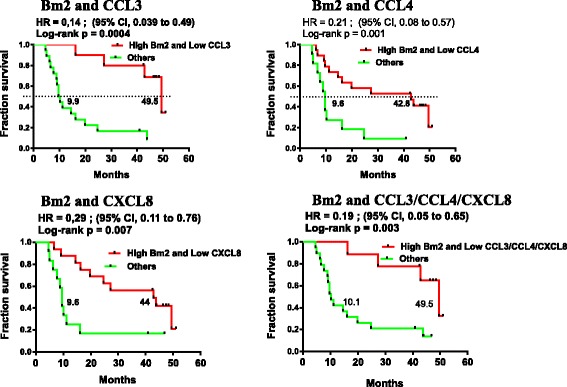
B cell subsets paired with chemokine levels correlate with OS. Kaplan-Meier OS curves comparing patients with high Bm2 population and low levels of listed chemokine (<67 pg/ml for CCL3, or <600 pg/ml for CCL4 or <76 pg/ml for CXCL8) versus all other patients (others). *P* values were calculated using the log-rank test and are shown on each graph

OS was evaluated in patients with favorable B cell differentiation (i.e. high Bm2) combined with low levels of at least one described chemokine. These groups demonstrated highly significant improvements in OS (Bm2^hi^/CCL3^lo^ HR = 0.14 (95%CI:0.04–0.49) *p* = 0.0004; Bm2^hi^/CCL4^lo^ HR = 0.21 (95%CI:0.08–0.57), *p* = 0.001; and Bm2^hi^/CXCL8^lo^ HR = 0.29 (95%CI:0.11–0.76), *p* = 0.007, Figure 5). Finally, patients with high levels of Bm2 and low levels of all 3 chemokines similarly displayed improved OS (HR = 0.19 (95%CI:0.05–0.65), *p* = 0.003).

Overall, data obtained from peripheral markers collected before any treatment show that increase of CCL3, CCL4, CXCL8 or advanced B lymphocyte differentiation status were strongly associated with worse clinical outcomes.

### Treatment was associated with changes in B cell differentiation status and an increase in antibody production

**Fig. 6:**
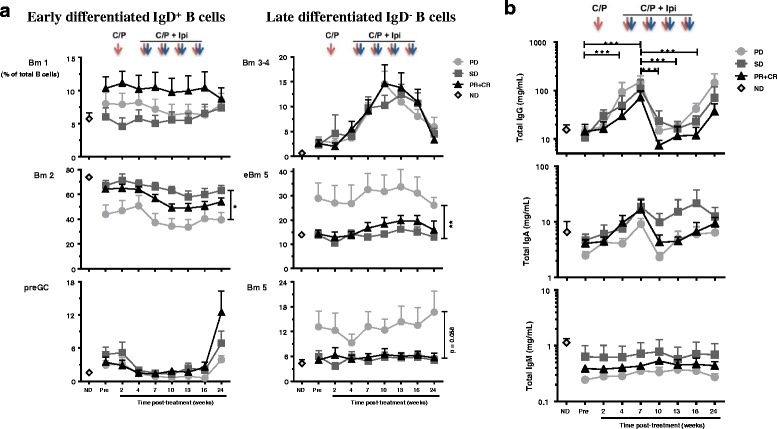
On treatment monitoring of circulating B lymphocyte differentiation status and Ig levels. **a** Circulating B lymphocyte differentiation status for each Bm population before and during treatment. Effect of response groups and time points, and their interaction were assessed with a one-way ANOVA group comparison using a linear mixed effects models for repeated measures; between indicated groups, *: *p* < 0.05; **: *p* < 0.01. **b** Evaluations of total circulating levels of IgG (top), IgA (middle) and IgM (bottom). A one-way ANOVA group comparison using a linear mixed effects models for repeated measures statistical analysis reveals that all groups had statistical variations in time for IgG; ***: *p* < 0.001

B lymphocyte differentiation status and circulating antibody levels were measured throughout the treatment. In both Bm2 and eBm5 + Bm5 subsets, the baseline difference observed in irPR + irCR compared to irPD patients was upheld throughout the study (from a linear mixed effect models analysis, *p* < 0.05 for Bm2, *p* < 0.01 for eBm5 and *p* = 0.058 for Bm5 respectively; Figure 6a). Conversely, study treatment was accompanied by a change in B cell subset composition across all patient response groups. The Bm3–4 subsets (sIgD^−^CD38^hi^), corresponding to centroblasts/centrocytes germinal center B cells, increased in all patients following initial exposure to ipilimumab (*p* < 0.05 for variation in time for all groups, Figure 6a). The Bm3–4 subset represented over 10% of B cells at week 10, compared to 3% before treatment (Fig. 6a). To investigate whether treatment had further influence on B cell activity, levels of circulating immunoglobulins were evaluated. Circulating IgG increased for all patient groups (*p* < 0.001), IgA also increased but was not statistically significant, while IgM levels remained unchanged (Figure 6b).

These results illustrate that B cell phenotype and function were affected in all patients receiving ipilimumab and CP. irPD patients maintained advanced B cell differentiation status throughout treatment.

### Ipilimumab exposure is followed by an increase in PD-1+ T cell subsets in non-responders

**Fig. 7:**
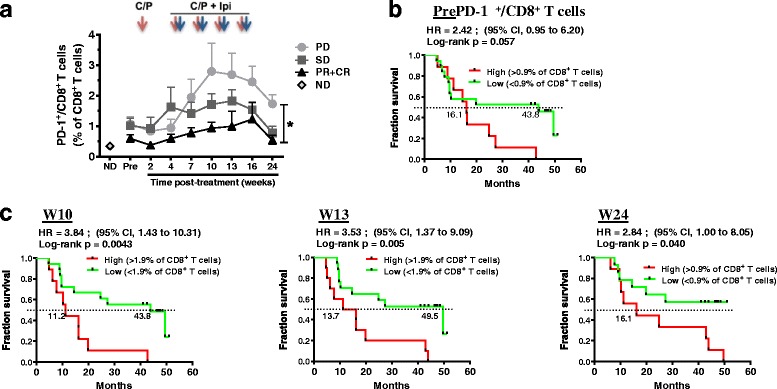
Monitoring of circulating CD8^+^PD-1^+^ levels and impact on OS. **a** Circulating CD8^+^PD-1^+^ T cell levels before and during treatment. Effect of response groups and time points and their interaction were assessed with a one-way ANOVA group comparison using a linear mixed effects models for repeated measures; *: *p* < 0.05 between irPD and irPR + CR groups. **b**-**c** Kaplan-Meier OS curves for the CD8^+^PD-1^+^ T cell population, stratified by mean percentages at baseline (**b**, Pre) and each indicated time point (**c**). *P* values were calculated using the log-rank test and are shown on each graph

The levels of selected surface markers related to T cell function were evaluated on circulating CD4^+^ and CD8^+^ T cells before and throughout treatment with ipilimumab and CP. In CD4^+^ T cells, a trend increase in the PD-1^+^ cells was observed following exposure to ipilimumab, although this apparent increase did not yield statistically significant correlations with response or survival (Additional file 1: Figure S5A). No discernable increase was observed in CD4^+^ Treg cells during treatment (Additional file 1: Figure S5B). Pre-treatment proportions of CD4^+^ Treg cells did not correlate with response or survival (Additional file 1: Figure S5B, Additional file 2: Table S5). In contrast, increased proportions of PD-1^+^CD8^+^ T cells were seen following ipilimumab exposure in patients with irPD (*p* < 0.05, Fig. 7). Although baseline proportions of PD-1^+^CD8^+^ cells were not associated with response (p = ns), responding patients had a significantly lower proportion of PD-1^+^CD8^+^ cells than patients with irPD at the end of the observation period (*p* < 0.05, Figure 7a). There was a trend in association between high levels of PD-1^+^CD8^+^ cells prior to start of treatment and worse OS (HR = 2.42 (95%CI:0.95–6.20), *p* = 0.057, Figure 7b). However, patients with higher proportions of these cells in circulation during treatment consistently had poorer OS (HR = 3.84 (95%CI:1.43–10.31), *p* = 0.004 at week 10; HR = 3.53 (95%CI:1.37–9.09), *p* = 0.005 at week 13 and HR = 2.84 (95%:1.00–8.05), *p* = 0.040 at week 24 (Figure 7c). A similar association between circulating PD-1^+^CD8^+^ T cells during treatment as a continuous variable and OS was observed (Additional file 2: Table S5).

ICOS expression was increased uniformly across all patient response groups on both CD4^+^ and CD8^+^ T cells following ipilimumab exposure, as previously observed for ipilimumab monotherapy (Additional file 1: Figure S6; top panels [7, 26, 27]). Increase in ICOS was not associated with response or OS (Additional file 1: Figure S6; lower panels, Additional file 2: Table S5).

## Discussion

This phase II study explored the toxicity, activity and predictive biomarkers of the combination of CP with ipilimumab. The study of this combination reveals manageable toxicity, with most patients completing the full course of treatment. IrAEs were consistent with previously published studies [2, 28]. Our study showed fewer grade 3/4 irAEs and no immune-related hepatotoxicity, when compared to ipilimumab-DTIC arm, where more than 33% of patients experienced grade 3 or more irAEs [3]. The difference in toxicity profile may be explained by the lower dose of ipilimumab used in this study or the use of prophylactic corticosteroids prior to paclitaxel infusions, thus potentially diminishing the severity of irAEs. A previous study showed higher hepatotoxicity when ipilimumab was combined with DTIC, rather than carboplatin/paclitaxel [28]. The performed comprehensive immune correlative study revealed new predictive circulating signatures linked to pre-existing inflammatory state associated with worse patient outcome.

The median OS (16.2 months) as well as 1-year OS (56.7%), 2-year OS (43.3%) and 3-year OS (36.7%) were comparable to previously published responses to ipilimumab in the first-line setting yet this regimen offers no survival benefit [3, 29–32]. Similarly to Weber et al. the observed ORR was 27% by irRC criteria [28]. The limitations of cross-study comparisons are recognized. Outcomes from the current study may have been influenced by the small patient population with mostly normal pre-treatment LDH, few patients with brain metastases and most patients with ECOG of 0. The criteria used to assess response (irRC versus RECIST 1.1) and the availability of additional systemic treatment options post-progression may have affected the results.

The impact of prophylactic corticosteroids use on anti-tumor immune responses with ipilimumab is poorly understood and a source of concern. Sequential treatment with ipilimumab and CP previously showed improved progression-free survival (PFS) in non-small cell lung carcinoma compared to concurrent treatment in a randomized phase II trial [33]. Patients on this current study were randomized in a 1:2 fashion favoring the sequential arm. Our results in melanoma suggest effective anti-tumor activity regardless of the scheduling of corticosteroid premedication (Additional file 2: Table S3).

Understanding mechanisms that influence patient response to immunotherapies will help shape future targeting strategies of the immune response. Aspects of the immune response were studied both systemically and within the tumor environment. The genetic composition of the tumor may ultimately impact on its immunogenicity. Although there is evidence that mutant *BRAF* could trigger immune responses in melanoma cells under certain contexts [34], our data are more consistent with a diminished chance of clinical benefit to ipilimumab in patients with *BRAF* mutations [35–37]. Recent subgroup analysis results from the CheckMate 067 demonstrated a favorable OS outcome for patients with *BRAF*-mutant tumors in the ipilimumab cohort in previously untreated patients [30]. 6/9 *BRAF* mutated patients had failed a BRAF inhibitor which may have contributed to poorer outcomes. One report demonstrated that patients with *NRAS* mutant metastatic melanoma achieved increased clinical benefit from ipilimumab compared to patients with *BRAF*/*NRAS* wild type melanomas [38]. We did not confirm these results in our study, since our three patients with *NRAS* mutant melanoma all had PD as BOR.

A higher immune score in pre-treatment tumor biopsies correlated with BOR. This correlation is in line with the predictive value of T cell infiltrates in regional nodal metastases and benefit in patients treated with neoadjuvant interferon-α-2b therapy [39, 40]. The presence of a strong immune cell infiltrate in the tumor microenvironment suggests that the tumor elicits T cell infiltration and that anti-tumor T cell activation can be enhanced by stimulating signals such as ipilimumab.

In contrast, we found that increased systemic inflammation at baseline is associated with poor response to therapy and poor OS. Non-responders displayed baseline differences in circulating chemokines CXCL8 and CCL4 in comparison to the levels of NDs and responders. CXCL8 is associated with chronic inflammatory states [41]. Moreover, circulating levels of CXCL8 have previously been associated with poorer prognosis in melanoma and other malignancies [42, 43], and an early increase in serum CXCL8 after initiation of anti-PD-1 treatment was associated with poor response and survival [44]. CCL4 and CCL3 may mediate the recruitment of CD8^+^ T cells and regulatory T cells within melanoma lesions [45]. In this trial, baseline elevated circulating CCL3, CCL4 and CXCL8, alone or in combination, were associated with substantially worse patient outcomes (Figs. 3 and 5), thus making them potential predictive biomarkers for patients most likely to fail treatment. The role of chemokines in mediating resistance to therapy remains to be investigated.

A specific skewing in B cell differentiation prior to treatment was noted in non-responders, with higher eBm5/Bm5 terminally differentiated B cells compared to responders and NDs (Figure 4). This also strongly correlated with OS. These results may reflect a general state of inflammation in non-responders as reported in other chronic conditions [46, 47]. B cell differentiation status was maintained throughout the study, suggesting that ipilimumab did not reverse this effect. Interestingly, Bm3–4 population, corresponding to centroblasts/centrocytes antibody producing B cells, increased with treatment in all patients, independently of clinical outcome. Study treatment had an effect on B cell biology, leading to fluctuations in circulating antibodies. CTLA-4 has been shown to modulate B cell responses through modulation of T follicular helper, T follicular regulatory, and T regulatory cells in animal models [48, 49]. This is the first evidence showing that ipilimumab treatment in humans affects B lymphocyte differentiation and function. However, the contribution of chemotherapy and the influence of systemic corticosteroids as prophylaxis remains to be established. The impact of the treatment regimen on OS of patients depended on the baseline B cell differentiation status, alone or in combination with chemokines. The impressive combined effect of baseline early B cell populations and chemokines on OS needs to be validated. Indeed, if these associations are confirmed, they might bring new predictive circulating biomarkers to immunotherapy.

Finally, an increase in the CD8^+^PD-1^+^ T cell population in non-responders and a robust impact on OS during treatment could be a sign of immune exhaustion and points to a possible escape mechanism to anti-CTLA-4. It is likely that simultaneous targeting of multiple immune checkpoints such as PD-1 and CTLA-4 [50] may be required to overcome compensatory mechanisms. This further suggests that additional negative immune regulators should be monitored after immunotherapies to identify relevant new targets to overcome resistance mechanisms.

## Conclusions

In summary, we report that ipilimumab and CP can be used in combination yielding manageable toxicities and favorable response, even if given concurrently. Our study revealed tumor-related and peripheral signatures associated with ipilimumab/CP resistance and OS. CD3^+^ T cell infiltration of the tumor correlated with good response, whereas the presence of a *BRAF* or *NRAS* mutation correlated with poor response, especially in patients pretreated with a BRAF inhibitor. Resistance to treatment was associated with pre-existing systemic inflammatory state, specifically elevated CCL4 and CXCL8, baseline B lymphocyte subset skewing, and increased CD8^+^PD-1^+^ T lymphocytes. From a comprehensive basic immune monitoring, we provide evidence for new predictive circulating biomarkers linked to OS. Larger studies are required to validate these circulating biomarkers. Future studies will address the safety of other checkpoint inhibitors combined with chemotherapy and determine whether biomarkers associated with improved outcomes to CTLA-4 directed therapy may be relevant to the clinical activity of inhibitors of other checkpoints.

## Additional files

Additional file 1: Supplemental Figures. **Figure S1.**  Kaplan-Meier curves. **Figure S2.** Evaluation of main immune cell population from samples collected before treatment (Pre). **Figure S3.** Evaluation of cell surface activation markers on CD4+ and CD8+ T lymphocyte subsets. **Figure S4.** Bm classification of B cell subsets. **Figure S5.** T cell subsets (CD4^+^ PD-1^+^ and Tregs) analysis throughout treatment. **Figure S6.** ICOS expression on T cells throughout treatment. (PPTX 653 kb)

Additional file 2: Supplemental Tables. **Table S1.** Demographic and baseline characteristics of the patients seperated by arm. **Table S2.** Adverse Events. **Table S3.** Tumor Response (by irRC, mWHO and Arm). **Table S4.** PD-L1 expression in the two Best Overall Response groups. **Table S5.** Univariate Cox regression models. **Table S6.** Antibodies for characterization of circulating immune cells. **Table S7.** Peripheral soluble cytokines/chemokines/soluble receptors studied by multiplex. (DOCX 50 kb)

## Abbreviations

AE: Adverse event
BOR: Best overall response
BORR: Best overall response rate
CBR: Clinical benefit rate
CP: Carboplatin paclitaxel
CR: Complete response
CTLA-4: Cytotoxic T-cell lymphocyte-associated protein 4
DCR: Disease control rate
ECOG: Eastern cooperative oncology group
irAE: Immune-related adverse event
irRC: Immune-related response criteria
LDH: Lactate dehydrogenase
MAPK: Mitogen-activated protein kinase
MDSC: Myeloid-derived suppressor cells
mWHO: Modified World Health Organization
ND: Normal donor
OS: Overall survival
PD: Progressive disease
PD-L1: Programmed death-ligand 1
PFS: Progression free survival
PR: Partial response
SAE: Serious adverse event; SD: Stable disease
