# Supplementary material for: Peripheral and local predictive immune signatures identified in a phase II trial of ipilimumab with carboplatin/paclitaxel in unresectable stage III or stage IV melanoma
Source: J Immunother Cancer. 2017 Nov 21;5:83. doi: 10.1186/s40425-017-0290-x (PMC5696743; doi:10.1186/s40425-017-0290-x)
Supplement: Supplementary file 1 — Figure S1. Kaplan-Meier curves. Figure S2. Evaluation of main immune cell population from samples collected before treatment (Pre). Figure S3. Evaluation of cell surface activation markers on CD4+ and CD8+ T lymphocyte subsets. Figure S4. Bm classification of B cell subsets. Figure S5. T cell subsets (CD4+ PD-1+ and Tregs) analysis throughout treatment. Figure S6. ICOS expression on T cells throughout treatment. (PPTX 653 kb) [file 40425_2017_290_MOESM1_ESM.pptx]

## Slide 1
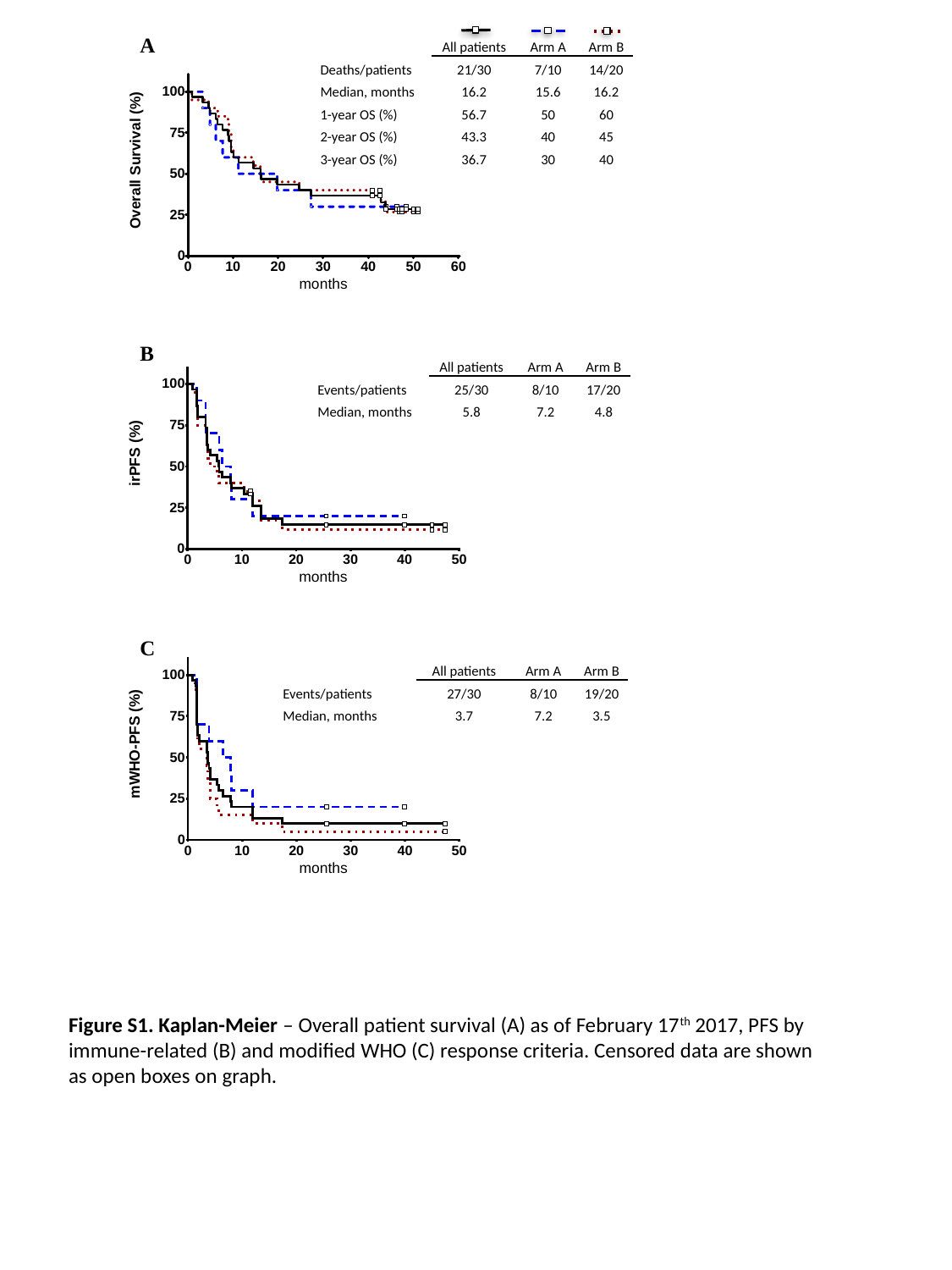

A
| | All patients | Arm A | Arm B |
| --- | --- | --- | --- |
| Deaths/patients | 21/30 | 7/10 | 14/20 |
| Median, months | 16.2 | 15.6 | 16.2 |
| 1-year OS (%) | 56.7 | 50 | 60 |
| 2-year OS (%) | 43.3 | 40 | 45 |
| 3-year OS (%) | 36.7 | 30 | 40 |
B
| | All patients | Arm A | Arm B |
| --- | --- | --- | --- |
| Events/patients | 25/30 | 8/10 | 17/20 |
| Median, months | 5.8 | 7.2 | 4.8 |
| | | | |
| | | | |
C
| | All patients | Arm A | Arm B |
| --- | --- | --- | --- |
| Events/patients | 27/30 | 8/10 | 19/20 |
| Median, months | 3.7 | 7.2 | 3.5 |
| | | | |
| | | | |
Figure S1. Kaplan-Meier – Overall patient survival (A) as of February 17th 2017, PFS by immune-related (B) and modified WHO (C) response criteria. Censored data are shown as open boxes on graph.

## Slide 2
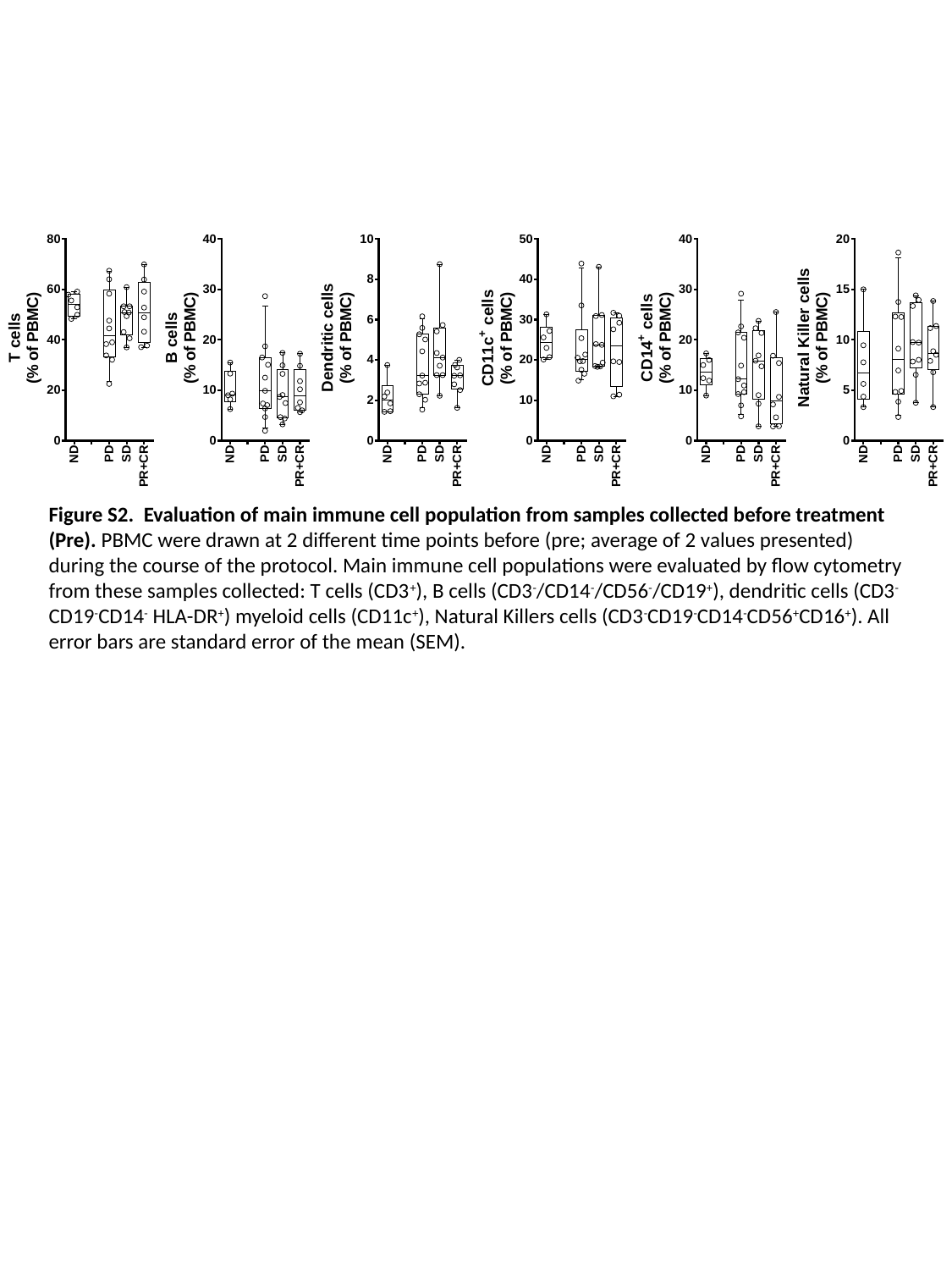

Figure S2. Evaluation of main immune cell population from samples collected before treatment (Pre). PBMC were drawn at 2 different time points before (pre; average of 2 values presented) during the course of the protocol. Main immune cell populations were evaluated by flow cytometry from these samples collected: T cells (CD3+), B cells (CD3-/CD14-/CD56-/CD19+), dendritic cells (CD3-CD19-CD14- HLA-DR+) myeloid cells (CD11c+), Natural Killers cells (CD3-CD19-CD14-CD56+CD16+). All error bars are standard error of the mean (SEM).

## Slide 3
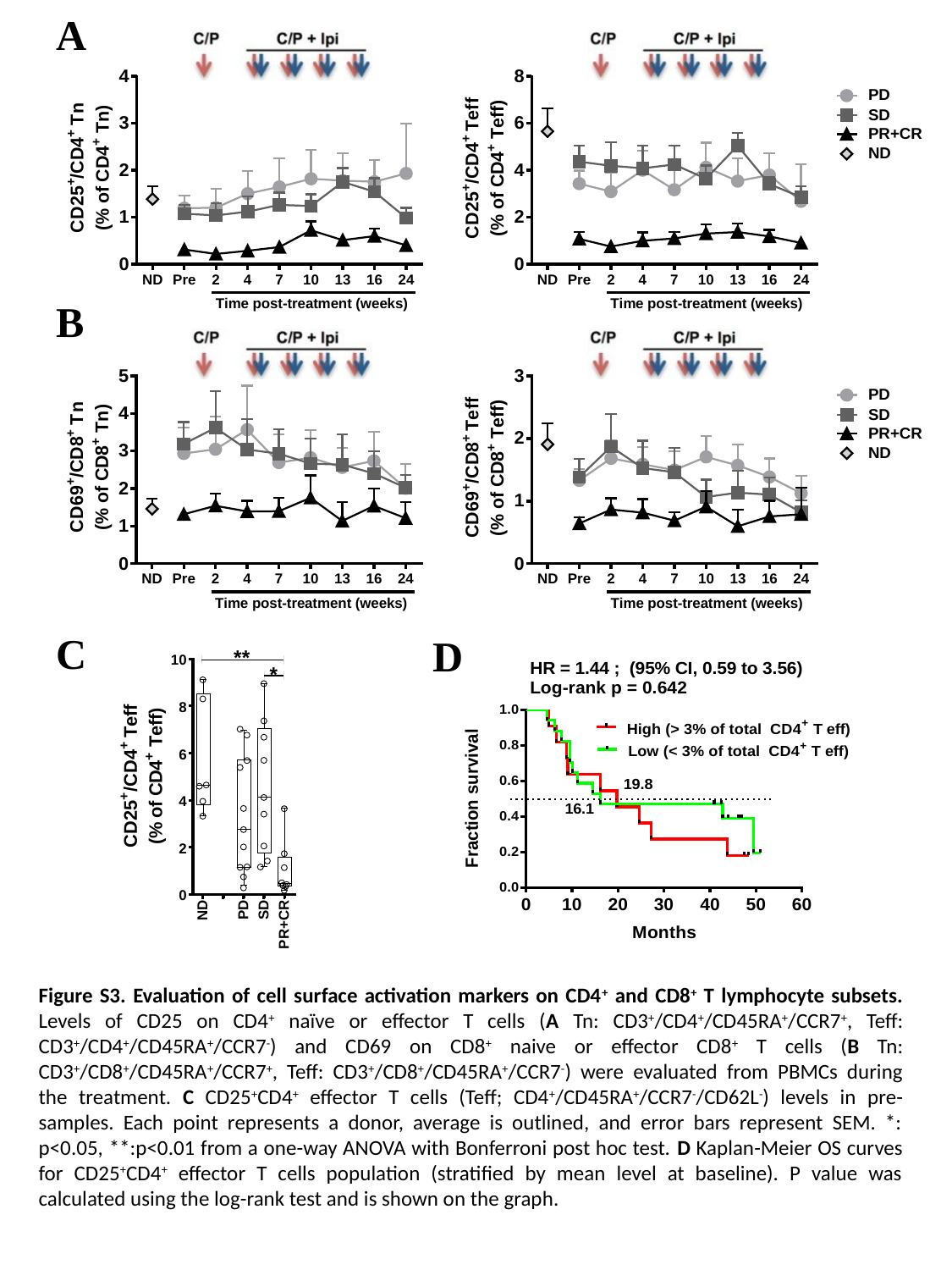

A
B
C
D
Figure S3. Evaluation of cell surface activation markers on CD4+ and CD8+ T lymphocyte subsets. Levels of CD25 on CD4+ naïve or effector T cells (A Tn: CD3+/CD4+/CD45RA+/CCR7+, Teff: CD3+/CD4+/CD45RA+/CCR7-) and CD69 on CD8+ naive or effector CD8+ T cells (B Tn: CD3+/CD8+/CD45RA+/CCR7+, Teff: CD3+/CD8+/CD45RA+/CCR7-) were evaluated from PBMCs during the treatment. C CD25+CD4+ effector T cells (Teff; CD4+/CD45RA+/CCR7-/CD62L-) levels in pre-samples. Each point represents a donor, average is outlined, and error bars represent SEM. *: p<0.05, **:p<0.01 from a one-way ANOVA with Bonferroni post hoc test. D Kaplan-Meier OS curves for CD25+CD4+ effector T cells population (stratified by mean level at baseline). P value was calculated using the log-rank test and is shown on the graph.

## Slide 4
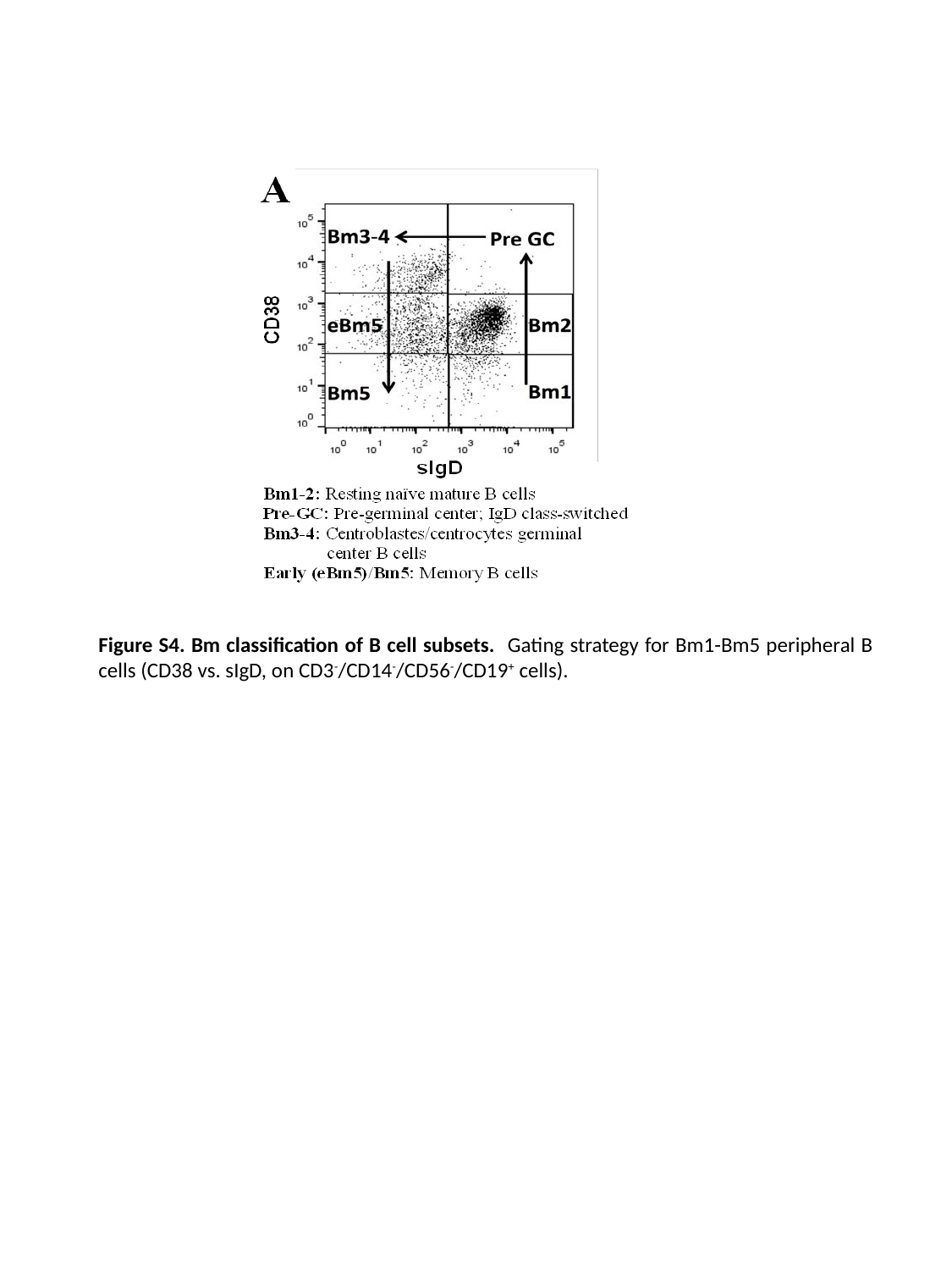

Figure S4. Bm classification of B cell subsets. Gating strategy for Bm1-Bm5 peripheral B cells (CD38 vs. sIgD, on CD3-/CD14-/CD56-/CD19+ cells).

## Slide 5
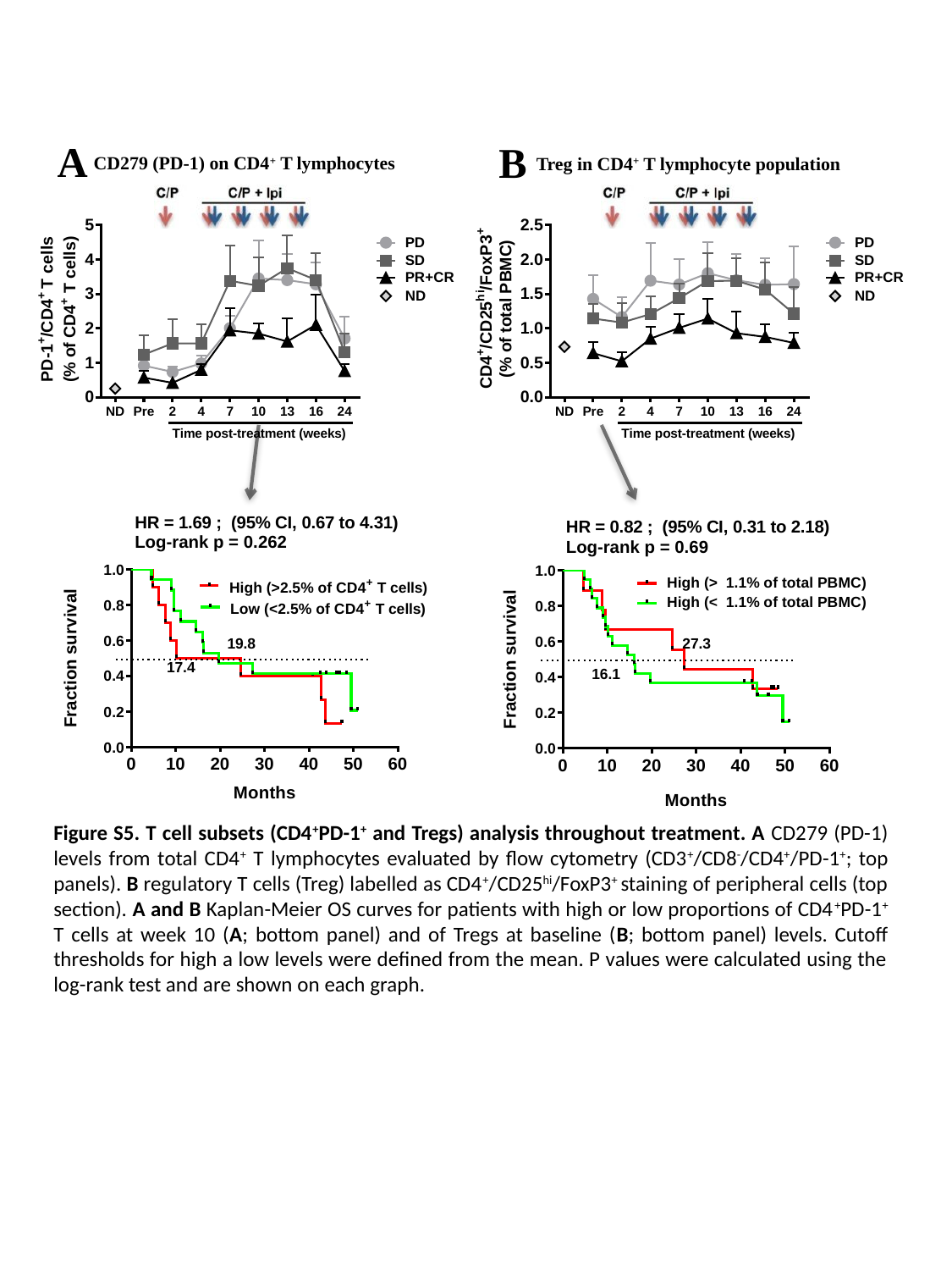

A
B
CD279 (PD-1) on CD4+ T lymphocytes
Treg in CD4+ T lymphocyte population
Figure S5. T cell subsets (CD4+PD-1+ and Tregs) analysis throughout treatment. A CD279 (PD-1) levels from total CD4+ T lymphocytes evaluated by flow cytometry (CD3+/CD8-/CD4+/PD-1+; top panels). B regulatory T cells (Treg) labelled as CD4+/CD25hi/FoxP3+ staining of peripheral cells (top section). A and B Kaplan-Meier OS curves for patients with high or low proportions of CD4+PD-1+ T cells at week 10 (A; bottom panel) and of Tregs at baseline (B; bottom panel) levels. Cutoff thresholds for high a low levels were defined from the mean. P values were calculated using the log-rank test and are shown on each graph.

## Slide 6
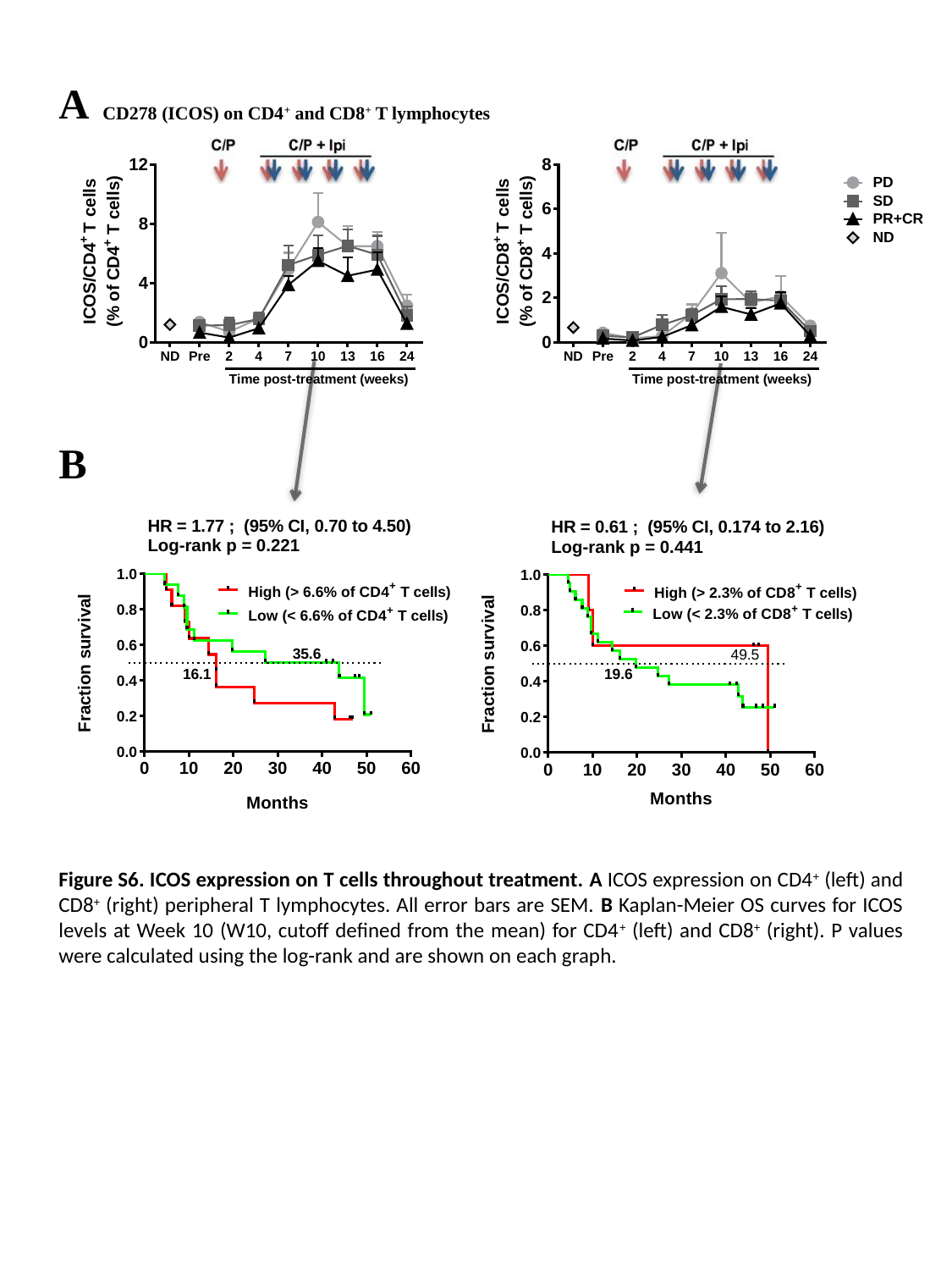

A
CD278 (ICOS) on CD4+ and CD8+ T lymphocytes
B
Figure S6. ICOS expression on T cells throughout treatment. A ICOS expression on CD4+ (left) and CD8+ (right) peripheral T lymphocytes. All error bars are SEM. B Kaplan-Meier OS curves for ICOS levels at Week 10 (W10, cutoff defined from the mean) for CD4+ (left) and CD8+ (right). P values were calculated using the log-rank and are shown on each graph.
