# Supplementary material for: Peripheral and local predictive immune signatures identified in a phase II trial of ipilimumab with carboplatin/paclitaxel in unresectable stage III or stage IV melanoma
Source: J Immunother Cancer. 2017 Nov 21;5:83. doi: 10.1186/s40425-017-0290-x (PMC5696743; doi:10.1186/s40425-017-0290-x)
Supplement: Supplementary file 2 — Table S1. Demographic and baseline characteristics of the patients seperated by arm. Table S2. Adverse Events. Table S3. Tumor Response (by irRC, mWHO and Arm). Table S4. PD-L1 expression in the two Best Overall Response groups. Table S5. Univariate Cox regression models. Table S6. Antibodies for characterization of circulating immune cells. Table S7. Peripheral soluble cytokines/chemokines/soluble receptors studied by multiplex. (DOCX 50 kb) [file 40425_2017_290_MOESM2_ESM.docx]

**A Phase II trial of Ipilimumab with Carboplatin/Paclitaxel in Unresectable Stage III or Stage IV Melanoma reveals peripheral and local immune signature**

Jamal et al.

Supplemental Tables

Supplemental Tables

Table S1. Demographic and Baseline Characteristics of the Patients Seperated by Arm (Number (percent))

| *Patient Characteristics* |  | *Arm A* | | *Arm B* | |
| --- | --- | --- | --- | --- | --- |
| **Total no. of patients** |  | **10 (33)** | | **20 (67)** | |
| **Median age, years (range)** |  | **55 (43-67)** | | **52.5 (26-74)** | |
|  |  |  | |  | |
| **Sex** |  |  | |  | |
| Female |  | **2 (20)** | | **6 (30)** | |
| Male |  | **8 (80)** | | **14 (70)** | |
|  |  |  | |  | |
| **Metastatic stage (n)** |  |  | |  | |
| M0 |  | **0** | | **1 (5)** | |
| M1a |  | **2 (20)** | | **4 (20)** | |
| M1b |  | **5 (50)** | | **1 (5)** | |
| M1c |  | **3 (30)** | | **14 (70)** | |
|  |  |  | |  | |
| **Lactate dehydrogenase** |  |  | |  | |
| ≤ ULN* |  | **7 (70)** | | **13 (65)** | |
| > ULN |  | **3 (30)** | | **7 (35)** | |
|  |  |  | |  | |
| **ECOG** |  |  | |  | |
| 0 |  | **6 (60)** | | **13 (65)** | |
| 1 |  | **4 (40)** | | **7 (35)** | |
|  |  |  | |  | |
| **Primary site** |  |  | |  | |
| Cutaneous |  | **10 (100)** | | **14 (70)** | |
| Mucosal |  | **0** | | **2 (10)** | |
| Ocular |  | **0** | | **3 (15)** | |
| Unknown primary |  | **0** | | **1 (5)** | |
|  |  |  | |  | |
| **BRAF status** |  |  | |  | |
| BRAF mutated (V600E) |  | **3 (30)** | | **6 (30)** | |
| BRAF wild type |  | **7 (70)** | | **14 (70)** | |
|  |  |  | |  | |
| **Prior therapies**** |  |  | |  | |
| Prior adjuvant therapy |  | **4 (40)** | | **1 (5)** | |
| Prior therapy with a BRAF inhibitor |  | **3 (30)** | | **3 (15)** | |
|  |  |  | |  | |
| **Brain metastases** |  |  | |  | |
| Patients without brain metastases |  | **7 (70)** | | **19 (95)** | |
| Patients with brain metastases |  | **3 (30)** | | **1 (5)** | |
|  |  |  | |  | |
| * ULN denotes upper limit of the normal |  |  | |  | |
| Table S2. Adverse Events | | | |  | |

|  | **ARM A (n=10)** | | **ARM B (n=20)** | |
| --- | --- | --- | --- | --- |
|  | **Total** | **Grade3/4/5** | **Total** | **Grade3/4/5** |
| **All adverse events** |  |  |  |  |
| Any event | 10 (100) | 6 (60) | 20 (100) | 15 (75) |
| Hepatotoxicity |  |  |  |  |
| high ALT | 2 (20) | 1 (10) | 2 (10) | 1 (5) |
| Gastrointestinal Disorders |  |  |  |  |
| nausea | 4 (40) | 0 | 17 (85) | 1 (5) |
| vomiting | 3 (30) | 0 | 11 (55) | 0 |
| diarrhea | 7 (70) | 0 | 16 (80) | 3 (15) |
| *C.difficile* colitis | 1 (10) | 1 (10) | 0 | 0 |
| Electrolyte |  |  |  |  |
| hypophosphatemia | 1 (10) | 0 | 3 (15) | 2 (10) |
| hypokalemia | 2 (20) | 0 | 4 (20) | 1 (5) |
| hypomagnesemia | 2 (20) | 0 | 6 (80) | 1 (5) |
| Hematological |  |  |  |  |
| anemia | 2 (20) | 0 | 2 (10) | 2 (10) |
| febrile neutropenia | 0 | 0 | 2 (10) | 2 (10) |
| neutropenia | 3 (30) | 2 (20) | 5 (25) | 3 (15) |
| thrombocytopenia | 2 (20) | 1 (10) | 4 (20) | 2 (10) |
| Nervous System |  |  |  |  |
| seizure | 0 | 0 | 2 (10) | 1 (5) |
| vasovagal reaction | 0 | 0 | 1 (5) | 1 (5) |
| Infection |  |  |  |  |
| pneumonia | 1 (10) | 1 (10) | 0 | 0 |
| Constitutional |  |  |  |  |
| fatigue | 6 (60) | 1 (10) | 11 (55) | 1 (5) |
| Vascular Disorder |  |  |  |  |
| pulmonary embolism | 0 | 0 | 1 (5) | 1 (5) |
|  |  |  |  |  |
| **Immune-related adverse events** |  |  |  |  |
| Any event | 8 (80) | 1 (10) | 18 (90) | 3 (15) |
| Gastrointestinal Disorders |  |  |  |  |
| diarrhea | 3 (30) | 0 | 8 (40) | 2 (10) |
| vomiting | 2 (10) | 0 | 4 (20) | 0 |
| autoimmune colitis | 0 | 0 | 1 (5) | 1 (5) |
| Endocrine |  |  |  |  |
| hypothyroidism | 1 (10) | 0 | 0 | 0 |
| pituitary disorder | 0 | 0 | 1 (5) | 0 |
| Skin Disorders |  |  |  |  |
| rash | 2 (20) | 0 | 5 (25) | 0 |
| pruritus | 3 (30) | 0 | 6 (30) | 0 |
| urticaria | 0 | 0 | 1 (5) | 0 |
| vitiligo | 0 | 0 | 1 (5) | 0 |
| erythroderma | 1 (10) | 0 | 0 | 0 |
| Constitutional |  |  |  |  |
| fatigue | 3 (30) | 1 (10) | 8 (40) | 0 |
|  |  |  |  |  |

Table S3. Tumor Response (by irRC, mWHO and Arm)

|  | All patients | Arm A | Arm B |  |
| --- | --- | --- | --- | --- |
|  | n=30 | n=10 | n=20 | A *vs.* B |
| Response | No. of pts (%) | No. of pts (%) | No. of pts (%) | P-value |
|  |  |  |  |  |
| **irBOR** |  |  |  |  |
| irCR | 1 | 0 | 1 |  |
| irPR | 7 | 2 | 5 |  |
| irSD | 9 | 5 | 4 |  |
| irPD | 13 (43) | 3 (30) | 10 (50) | 0.18 |
| irDCR | 17 (57) | 7(70) | 10 (50) | 0.43 |
| irCBR | 13 (43) | 6 (60) | 7 (35) | 0.23 |
| irBORR | 8 (27) | 2 (20) | 6 (30) | 0.29 |
|  |  |  |  |  |
| **mWHO-BOR** |  |  |  |  |
| mWHO-CR | 0 | 0 | 0 |  |
| mWHO-PR | 4 | 2 | 2 |  |
| mWHO-SD | 7 | 4 | 3 |  |
| mWHO-PD | 19 (63) | 4 (40) | 15 (75) | 0.11 |
| mWHO-DCR | 11 (37) | 6 (60) | 5 (25) | 0.09 |
| mWHO-CBR | 8 (27) | 6 (60) | 2 (10) | 0.01 |
| mWHO-BORR | 4 (13) | 2 (20) | 2 (10) | 0.15 |
|  |  |  |  |  |
| *Abbreviations: BOR, best overall response; ; ir, immune-related; mWHO, modified WHO; CR, complete response; PR, partial response; SD, stable disease; PD, progressive disease; BORR, best overall response rate; DCR, disease control rate (CR+PR+SD); CBR, clinical benefit rate (CR+PR+SD≥24 weeks)* | | | | |

## Table S4. PD-L1 expression in the two Best Overall Response groups


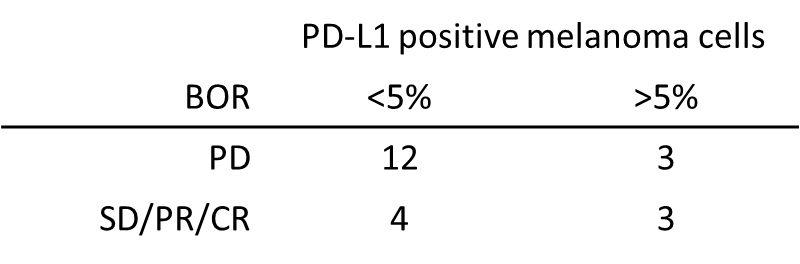


No correlation between the percentage of PD-L1 positive melanoma cells and BOR (Pearson Chi-Square 1.257, 2-sided Fisher Exact Test 0.334)

## Table S5. Univariate Cox regression models

| Variables | P value | HR (95% CI) |
| --- | --- | --- |
| PD-L1 | 0.920 | 0.998 (0.954 – 1.044) |
| CCL3 | 0.035 | 14.144 (1.211 – 165.249) |
| CCL4 | 0.001 | 5.941 (2.062 – 17.120) |
| CXCL8 | 0.236 | 1.866 (0.665 – 5.235) |
| Bm2 | 0.007 | 0.113 (0.023 – 0.549) |
| eBm5+Bm5 | 0.027 | 10.268 (1.302 – 80.959) |
| Pre PD1^+^/CD8^+^ | 0.057 | 1.792 (0.983 – 3.268) |
| W10 PD1^+^/CD8^+^ | 0.002 | 1.444 (1.141 – 1.827) |
| W13 PD1^+^/CD8^+^ | 0.001 | 1.528 (1.178 – 1.982) |
| W24 PD1^+^/CD8^+^ | 0.007 | 2.518 (1.291 – 4.911) |
| Pre CD25^+/^CD4^+^ Teff | 0.581 | 1.045 (0.893 – 1.223) |
| W10 PD-1^+^/CD4^+^ | 0.743 | 1.029 (0.867 – 1.221) |
| Pre Treg/CD4^+^ | 0.363 | 1.267 (0.761 – 2.108) |
| W10 ICOS^+^/CD4^+^ | 0.214 | 1.070 (0.962 – 1.119) |
| W10 ICOS^+^/CD8^+^ | 0.438 | 1.048 (0.932 – 1.178) |

**Cox regression models: significance p<0.05 and hazard ratio (HR) are indicated. CI : Confidence Interval**

## Table S6. Antibodies for characterization of circulating immune cells

| Antibodies | Fluorochromes | Antibodies | Fluorochromes |
| --- | --- | --- | --- |
| CD3 | FITC | CD45RA | FITC |
| CD3 | Alexa Fluor 700 | CD56 | FITC |
| CD4 | FITC | CD62L | PE-CF594 |
| CD4 | V450 | CD66b | PE |
| CD8 | APC-H7 | CD69 | PerCPCy5.5 |
| CD11c | APC-H7 | CD80 | APC-H7 |
| CD11c | V450 | CD86 | Alexa Fluor 700 |
| CD14 | FITC | CD127 | PE-CF594 |
| CD14 | PerCPCy5.5 | CD152 | APC |
| CD16 | PE-CF594 | CD206 | FITC |
| CD19 | PE | CD274 | PE-Cy7 |
| CD19 | PerCPCy5.5 | CD278 | PE |
| CD20 | APC-H7 | CD279 | PE |
| CD23 | APC-H7 | CCR7 | PE-Cy7 |
| CD24 | PE-Cy7 | sIgD | PE-CF594 |
| CD25 | APC | HLA-DR | PE-Cy7 |
| CD25 | PerCPCy5.5 | FoxP3 | V450 |
| CD27 | Alexa Fluor 700 | IFN-γ | PE-CF594 |
| CD33 | APC | Granzym B | FITC |
| CD38 | PE | IL-4 | APC |
| CD43 | APC | IL-17A | PE |

## Table S7. Peripheral soluble cytokines/chemokines/soluble receptors studied by multiplex.

| Cytokines | Chemokines | TNF Family |
| --- | --- | --- |
| IL-1β IL-2 | CXCL8 | TNF-α |
| IL-4 IL-5 IL-6 | CXCL9 | TNF-RIs |
| IL-7 IL-10 IL-12p70 | CXCL10 | TNF-RIIs |
| IL-13 IL-17 | CCL3 |  |
| IFN-γ | CCL4 |  |
| GM-CSF | CCL5 |  |
|  | VEGF |  |
